# Historical Account of the Fœtid Gums, and the Virtues Ascribed to Them

**Published:** 1809-06

**Authors:** 


### *Historical Account of the fœtid Gums, and the Virtues ascribed to them.*

### ASSAFŒTIDA.

THIS plant, of the natural order of Umbellata, and according to the sexual system of the order and class Pentandria Digynia, is of the genus Færula in Linnæus' Systema Vegetabilium. Its leaves are said to branch like the piony, but its external appearance will be considered hereafter. The gum-resin is said to drop from the roots properly cut in appearance almost milky, but by the heat of the sun it soon dries so as to become solid. When fresh, the smell is said to be so much more powerful than in its dried state, as to be absolutely insupportable to those who are not habituated to work at collecting it. The juice flows much more copiously from roots growing in the plain than on the mountains, and also from older than younger shoots. Those selected are of about four years growth, and in size equal to a man's leg at the calf. The root, after being cleansed from the mould that adheres to it, is reserved for more than a month before it is cut into transverse segments. From these segments, plenty of juice arises, which is received on a broad surface: as it dries, it is scraped off and received in baskets made of very delicate twigs. After a few days, the section is repeated with the same process in succession, and afterwards till the root is found to yield no more juice. This process we learn from Kaempfer, whose authority in these matters stands deservedly high. Bontius informs us, that in Persia, from whence we receive the drug, there are two kinds of assafœtida, one pro-

 cured cured in the manner we have described, the other from a juice pressed from the stem and leaves. This account has not, that I know, been confirmed.

It is by no means certain, that the ancient physicians, whose writings remain with us, were acquainted with this valuable medicine. Yet, considering the great intercourse between Persia and the rest of the civilized world, it seems difficult to conceive that an article of such general utility should have been unknown. The controversy on this subject would scarcely be interesting: but it may be worth while to remark, that Bauhine, Bodæus, Geoffrey, Millar, and Speilman, have written very learnedly and elaborately on the subject. Millar goes so far as to ascertain to what part of the plant, or to what particular preparations of the juice the Laser or Lasarpetium of Pliny and Celsus refer. Pliny recommends his laser in linctus's, or sippets for coughs. If this is really the same plant, it shows a surprising coincidence in the virtue of remedies according to their sensible properties; for there cannot be a doubt that garlick is one of the most powerful pectorals we are acquainted with, and also that it produces considerable effect on the urinary passages, for which Galen recommends laser. It is to be much regretted that the vulgar names of plants should be every where so uncertain. The word laser gives us no etymological assistance. The Arabic name of the plant is althit, the word assafœtida seems derived from the monks of the Salernian school.

But let us attend to what is better ascertained. Assafœtida is brought to us in pieces of various sizes and shape, of a mixed colour, partaking of brown, yellow, reddish, intermixed with small patches of white, somewhat transparent. The best is esteemed that which has a pale red complexion intermixed with white spots. When pressed between the teeth, it is tenacious enough to remain unbroken. Its smell is alliaceous, mixed with another twang, if possible, still less grateful. In large quantities, this is said to be so powerful, that the sailors have sometimes suspended the packages at the mast-head in their own defence, and to protect the rest of the freight. The taste is bitter and somewhat acrid. It suffers much with age, and is often greatly adulterated.

The experiments of Newman prove that it contains more gum than resin. He drew from it $\frac{1}{64}$ of essential oil, which, as may be conjectured, was extremely odorous. The water as well as spirit is much imbued with its smell by distillation. Pringle informs us, that the resin diffused in water has an antiseptic property. One should imagine that he scarcely made his experiments on esculent substances; but certain it is, that powerful as the smell may be, the Indians use it as a condiment for their food, rubbing with it the vessels in which they boil their broth, and even the edges of their bowls. This has, I believe, been sometimes imitated by some of our English epicures. But among the orientalists, the virtues of assafœtida as a remedy stand very high. The seeds themselves, though in a less degree, are esteemed in colic, in dropsy, and in tympanites.

tympanites. Externally, they use the fresh juice to cicatrize wounds. As a vulnerary, I can myself affirm, it possesses such powers, that a surgeon in this town conceived he had found in assafœtida a cure for cancer. Though in this, like many others, he was deceived, yet he certainly brought many ill conditioned ulcers to a kindly suppuration.

Among the Europeans, its virtues seem principally ascribed to its powerful odour. Whatever the immediate cause may be, it cannot be denied that the most honest and industrious practitioners have been the readiest at extolling its efficacy. Boerhaave does not scruple to say, that nothing is more powerful in nervous and spasmodic diseases. In this he is confirmed by Whytt, Forestus, Millar, Wolf, Plenck, and a tribe of others, who all unite in considering it as the most valuable resource in Hysteria, particularly for those symptoms which seem to arise from the state of the stomach during the remission of the paroxysm. In the acute spasmodic asthma of children, it has been found highly efficacious, where evacuants had been so far premised as to leave reason to suppose that no inflammatory action remained. In such cases, clysters are the only form in which it can be exhibited in sufficient quantity. In this form likewise, it has been recommended as an anthelmintic. But there is too much reason to believe that this opinion arose from the mere supposition that worms would as much dislike the smell of assafœtida as the patient. However this prejudice, like many others, has led to an useful practice, for without doubt assafœtida clysters are found efficacious in many of those anomalous complaints, which, for want of better knowledge, are conjectured to arise from those animals.

It was formerly very much the custom to add assafœtida to those purging pills to which in the decline of life men of sedentary habits are obliged to have recourse. Perhaps we have done wrong in omitting it, not only because a certain degree of hypochondriasis often attends this costiveness, but because the stimulus from assafœtida being more general, was less likely to leave the bowels costive. But we are not to estimate the virtues of this plant merely by these uncertain effects. Bergius assures us he has seen tympanites cured by a repetition of clysters three times a day, and that the pills taken during the remission of intermittents of long standing, have very often prevented the paroxysm altogether. The same author asserts, that a single grain of the fresh gum spreads an odour equal to 100lb. of the dried; and that the Banian Indians, who, as was before remarked, make it their principal condiment, use it also as their chief cordial and remedy for indigestion. That from such continual use, not only their breath, but their perspiration is continually tainted with its effluvia. This last is a certain proof that the substance circulates without any material change, through every part of the body; and when we consider the sensibility of the living fibre, it is not requiring too much to believe that spasms arising from mere irregular actions in them, may be arrested by so powerful an application.

Dr. Woodville's drawing is copied from Dr. Hope's paper, published in the Philosophical Transactions. It differs considerably from Kæmpfer's. No doubt can be entertained of the accuracy of Dr. Hope, whose drawing was taken from the growing plant. But it is not so certain that his was the true plant. Kæmpfer saw the whole process of extracting the gum, but I have not heard that Dr. Hope has procured the gum from his plant. Assafœtida enters into the composition of Pilul. Gum. or Galban. c. Tinct. fœtida, and spiritus ammoniæ fœtidus.

( To be continued. )

